# Smoking beliefs across genders, a comparative analysis of seven European countries

**DOI:** 10.1186/s12889-019-7700-6

**Published:** 2019-10-21

**Authors:** Adeline Grard, Michael Schreuders, Joana Alves, Jaana M. Kinnunen, Matthias Richter, Bruno Federico, Anton Kunst, Luke Clancy, Vincent Lorant

**Affiliations:** 1 Institute for Health and Society, University Catholic of Louvain, 30 clos chapelle-aux-champs, bte. L0.30.15, 1200 Woluwé-saint-Lambert, Brussels, Belgium; 2 Department of Public Health, University of Amsterdam, Amsterdam UMC, Amsterdam, the Netherlands; 3 Escola Nacional de Saúde Pública, Universidade NOVA de Lisboa, Lisboa, Portugal; 4 Faculty of Social Sciences, Health Sciences, University of Tampere, Tampere, Finland; 5 Institute of Medical Sociology, Faculty of Medicine, Martin Luther University Halle-Wittenberg, Halle, Germany; 6 Department of Human Sciences, Society and Health, Università degli studi di Cassino e del Lazio Meridionale, Cassino, Italy; 7 Tobacco Free Research institute, Dublin, Ireland

**Keywords:** Gender, Adolescent girls, Adolescent boys, Smoking beliefs, Smoking prevention, Adolescent smoking, Smoking prevention program, Gender-specific interventions

## Abstract

**Background:**

Most European countries have seen a decrease in the prevalence of adolescent smoking. This decrease has, however, been patterned by gender. Girls’ smoking rates have now overtaken boys’ in many European countries. The two genders may not, however, share the same smoking beliefs and this could explain differences between the genders in smoking prevalence. We describe gender differences in smoking beliefs and investigate variations between countries, along with their gender context.

**Methods:**

In 2016, we conducted the SILNE R study (Smoking Inequalities Learning from Natural Experiments – Renew) in 55 schools located in seven European countries: Belgium, Italy, The Netherlands, Portugal, Finland, Ireland, and Germany. We surveyed 12,979 students aged 14–16 years (50% were girls). We classified smoking beliefs into four categories: positive individual, positive social, negative individual, and negative social beliefs. We expected girls to score higher on the last three of those categories and we hypothesized that countries with a more gender-equal culture would have less gender difference in beliefs about smoking.

**Results:**

One out of two smoking beliefs differed significantly between genders. Negative social beliefs were more common in girls, while beliefs about the dating-related aspects of smoking were more common in boys. We identified Germany and Belgium as the only countries with no gender differences in any of the belief scales. No correlation was found, however, between these scales and the Gender Inequality Index.

**Conclusions:**

In some countries, gender-specific interventions might be implemented; however, two opposing strategies might be used, depending on whether such programs are aimed at boys or girls.

## Background

### Adolescent smoking prevention

Over the last two decades, most European countries have seen a decrease in the prevalence of adolescent smoking. This decrease has, however, been patterned by gender: in one out of two countries, most of them in southern and northern Europe, boys no longer smoke more than girls. In some western European countries, moreover, smoking rates among girls now surpass those among boys [1]. These prevalence trends suggest that the response to smoking prevention programs addressing adolescent smoking is patterned by gender. Some studies have suggested adapting smoking prevention strategies to gender contexts [2]. Most policy programs dedicated to the prevention of smoking in adolescents address smoking beliefs [3, 4], because smoking belief is associated with smoking status [5]. These prevention programs aim to either deconstruct positive smoking beliefs or reinforce negative attitudes to smoking. Girls and boys, however, may respond differently. In this article, we aim to compare smoking beliefs among European adolescents and to see how they vary between genders and countries. This should eventually help to better tailor interventions related to smoking beliefs for boys and for girls.

### Smoking beliefs

Several studies have focused on smoking beliefs among young people. One difficulty, however, is that they describe different beliefs, which are not always comparable. Some authors have highlighted the prevalence of social beliefs, about being popular or having fun with peers [6, 7], while others have focused on beliefs about the effects of tobacco, such as stress management [8]. Some of these studies, moreover, are old and/or only apply to the context of a specific country, whereas it is worth considering that smoking beliefs may have changed over time and may vary between countries. Nonetheless, following the current literature, smoking beliefs can be classified as promoting either positive or negative aspects of smoking and as relating to either individual or social concerns [9]. In the following paragraphs, we will briefly examine gender differences in the four categories of smoking belief.

#### Positive individual beliefs

Several studies have focused on two positive individual beliefs about smoking. On the one hand, in adolescence, tobacco consumption is used to cope with mood-related problems more frequently by girls than by boys [7, 10, 11]. One the other hand, girls are more receptive to the impact of smoking on weight reduction [12–15]. These studies suggest that positive individual beliefs are more often held by girls than by boys.

#### Positive social beliefs

Smoking is also often associated with pleasure and activities with friends [16], which are, however, more frequently cited by boys than by girls [7]. Moreover, adolescents think that smoking enhances peer conformity [8] and popularity [17–20], however, there is no difference in these beliefs between genders [8]. Furthermore, during adolescence, boys and girls are under more social pressure to adhere to gender roles [21, 22]. Smoking may help one to look grown-up or masculine (bad boy) or feminine (sophisticated, sexy). In Rugkasa’s study [23], smoking among girls was frequently associated with being attractive to boys, while smoking among boys was related with appearing grown-up or like hard men [23]. According to Lucas and Lloyd [24], however, smokers of both genders might smoke to be more attractive to the other gender.

#### Negative individual beliefs

Nevertheless, adolescents are nowadays well aware of the reasons not to smoke [25]. Lundborg [26] found that girls were more concerned about the health risks of smoking than boys. Moreover, girls were also more likely to quote smoking-related concerns about their physical appearance than boys [11, 27]. Boys, on the other hand, were more likely to consider athletic performance a reason to quit smoking [27]. Thus, while girls are more likely to identify with reasons not to smoke that are related to health and physical appearance, boys may be more sensitive to arguments related to physical activity.

#### Negative social beliefs

With smoking increasingly considered a deviant behavior, the way that significant peers sanction smoking could be a meaningful consideration for adolescents. For example, in a study by Riedel [27], both genders quoted other students’ negative attitudes to smoking as a reason not to smoke (13% girls; 14% boys). In Grogan [14], however, this was more frequently quoted by girls than by boys. With the increase in tobacco control policies, adolescents can also get into trouble, for instance if they are caught smoking at school, where it is forbidden. This should reduce the social attractiveness of smoking [28]. Nonetheless, for adolescents, transgression of a law could also be a reason to smoke, particularly among male peer groups [28]. It is widely acknowledged that boys are more often involved than girls in deviant behaviors, including substance use [29], while girls tend to display more rule-oriented behavior [30]. Thus, girls may be more sensitive to negative social aspects of smoking.

### Gender equality and health behavior

Differences in smoking beliefs between boys and girls might also depend on the level of gender inequality within their country. A recent study among adolescents found a positive association between the gender equality of a country’s culture (Gender Index of Inequality, GINI) and life satisfaction among adolescents [31]. A less masculine national culture was also found to be associated with less deviant behavior, such as bullying [1], and better mental health outcomes in adolescents [32, 33]. In relation to smoking, a study by Kuntsche [34] found a direct correlation between gender equality in the culture of a country and the female-to-male smoking ratio: the more gender-equal the country, the fewer differences there were in smoking rates between genders in adults [34, 35]. This correlation might be explained by the fact that, in countries with gender-equal cultures, women both have greater financial resources to access tobacco or other substances and are targeted more by industries that promote images of independence and empowerment associated with smoking. We might thus expect girls in such equal countries to have more positive beliefs about smoking [34–36]. To our knowledge, however, no study has addressed the association between the gender equality of a country’s culture and gender differences in smoking beliefs among adolescents.

### Knowledge gap

Concluding from the literature summarized above, we acknowledge that there is a lack of studies which systematically address gender differences in a broad range of smoking beliefs. Here, we will therefore address gender differences in a broad range of beliefs, which are classified according to the typology presented above. Moreover, many previous studies have used national samples. We will thus use an international sample, which allows us to investigate the extent to which both smoking beliefs and gender inequality are imbedded within their context. Ultimately, a deeper understanding of these factors might help to further improve and tailor smoking prevention programs. It could also be helpful in considering beliefs from an international perspective and could contribute to making such programs transferable from one context to another or, instead, tailoring them to the context of a specific country.

### Hypothesis

In this article, we hypothesized (1) that smoking beliefs among adolescents differ according to gender. We expected girls to score higher on positive individual, negative individual (except for physical activity), and negative social beliefs (see Table 1). We expected no gender differences in the social-status improvement beliefs (positive social beliefs). Secondly, we expected (2) gender differences in smoking beliefs to differ between countries. We hypothesized that countries with a more gender-equal culture would have less gender difference in beliefs, particularly social (positive and negative) beliefs about smoking, which should be more sensitive to contextual patterns than individual beliefs.

**Table 1:** Typology of smoking beliefs: expectations of gender patterns according to the literature

	Positive	Negative
Individual	Feeling relaxed (G) Losing weight (G)	Health risks (G) Physical appearance (G) Physical activity (B)
Social	Social influence (G) Social status/popularity (B&G) Attractiveness (B&G)	Friends’ influence (G) Getting into trouble (G)

Note: B: expected to be more frequently quoted by boys; G: expected to be more frequently quoted by girls

## Methods

In 2016–2017, we carried out a paper-and-pencil survey in 55 schools located in seven European cities: Namur (Belgium), Dublin (Ireland), Tampere (Finland), Hanover (Germany), Latina (Italy), Amersfoort (The Netherlands), and Coimbra (Portugal). This survey was the second wave of a cross-sectional study conducted in 2013. In each country, research teams selected a single city with a medium-range socioeconomic situation, and sampled six to eight high schools in order to (1) reach a sample of 2000 students per city and (2) be representative of the socioeconomic situation of the city. About 81% of schools agreed to participate in the survey again in 2016/17. When schools refused to participate again, we sampled new schools with the same socioeconomic background. As Ireland was not included in the first wave of the survey, we sampled eight new schools in the 2016 survey. The design used in both surveys has been described elsewhere [37]. Among other subjects, each student was asked about his/her beliefs related to smoking and about sociodemographic information. However, as smoking beliefs were the subject of a new question in the 2016/17 questionnaire, this paper only uses data collected in 2016/17.

In each classroom where data were collected, we used the official students’ register and carefully reported on the number of adolescents who (1) participated in the survey, (2) were absent, or (3) refused to participate. Participation rates were calculated as the percentage of adolescents registered in the classroom that actually participated in the survey. Participation rates were calculated in each classroom, then summed up at the school level and the country level. A total of 12,979 students aged 14–16 filled in the questionnaire in 2016/2017. For the whole sample, we achieved 79.9% participation, with slight differences between countries: Belgium (89%), Finland (89%), Ireland (81%), The Netherlands (89%), Portugal (88%), Italy (89%), and Germany (62%). Ethical approval was obtained for the second survey in all cities (see Acknowledgments); active written parental consent was requested and obtained in Germany and Italy, which might explain lower participation rates in Germany, and passive written parental consent was obtained in other countries. All adolescents, moreover, agreed to participate via written consent.

### Measurement

In this article, we develop an easy-to-use typology of beliefs, with two dimensions. The first dimension is the positive-negative opposition: *does this belief promote smoking (positive) or work to avoid its negative effects (negative)?* The second dimension of our typology is derived from the opposition between intrinsic and extrinsic beliefs about smoking cessation [9]. It is composed of, on the one hand, individual (intrinsic) beliefs, which come from the individual, and on the other hand, social (extrinsic) beliefs, which result from the social environment.

To measure smoking beliefs, we used a scale based on that of Song et al. [38], who used three factors that identify the perceived benefits, long-term risks, and short-terms risks of smoking. We selected and adapted this scale, removing some redundant items related to the health risks and disadvantages of tobacco, on which, it has been shown, there is about 100% agreement. Moreover, as we were interested in the social versus the individual dimension of smoking, we included additional social (looking grown-up, looking masculine, looking feminine, appearing sexy/attractive, getting a boy/girlfriend) and individual items (losing weight or staying thin, having less ability to exercise). Then, to add some complementary items to the negative social beliefs dimension, which only contained “getting into trouble” in Song et al., we used two items from the stigmatization scale [39]. We slightly adapted these to the context of adolescents: “Most people would not hire a smoker to take care of or babysit their children” and “Most smokers would be reluctant to date someone who smokes”. These items were selected because they were expected to vary according to gender, because boys and girls differ in their dating processes and involvement in babysitting [40].

First, the positive individual belief scale contains the following beliefs: “Do you think that smoking increases your chances of feeling relaxed?” and “Do you think that smoking increases your chances of losing weight or staying thin?” (Spearman-Brown coefficient: 0.44).

Second, the positive social belief scale was composed: of “Do you think that smoking increases your chances of becoming popular?”, “Do you think that smoking increases your chances of looking grown-up?”, “Do you think that smoking increases your chances of looking masculine?”, “Do you think that smoking increases your chances of looking feminine?”, “Do you think that smoking increases your chances of appearing sexy or attractive?”, and “Do you think that smoking increases your chances of getting a boyfriend or girlfriend?” (Cronbach alpha: 0.78).

Third, we computed the negative individual belief scale based on: “Do you think that smoking increases your chances of getting lung cancer?”, “Do you think that smoking increases your chances of having chronic trouble breathing?”, “Do you think that smoking increases your chances of getting facial wrinkles?”, and “Do you think that smoking increases your chances of having less ability to exercise?” (Cronbach alpha: 0.70).

Finally, we used two items from the stigmatization scale presented above (“Most people would not hire a smoker to take care of or babysit their children” and “Most smokers would be reluctant to date someone who smokes”) with the belief “Do you think that smoking increases your chances of getting into trouble?”, and also added friends’, parents’, and teachers’ disapproval of smoking (e.g. “My parents (would) disapprove if I smoked”, to create the negative social belief scale (Cronbach alpha: 0.45).

For each belief, we asked students to state whether they agreed that smoking was associated with the belief (e.g. “Do you think that smoking helps you to get a boy/girlfriend?”: “Totally agree”; “agree”; “don’t agree”; “totally disagree”). Each belief was then binarized into two categories (agreed (=1 point) or not (= 0 point) with the belief), which allowed us to describe the percentage of agreement with each belief. As the four belief scales contain different numbers of beliefs, we computed the arithmetic mean, in order to scale them. Then, we reported the total score of each on a 0–1 scale to use them as dependent linear variables. The beliefs were analyzed according to gender and country.

Gender was measured using the following question: “Are you a male or a female?”. Gender distribution was balanced in the total sample (50% of boys). The gender balance varied slightly between countries: 45% boys (Italy), 48% (Ireland) 50% (Belgium, Germany, and The Netherlands), 51% (Portugal), and 52% (Finland).

As smoking beliefs tend to differ across smoking status, we stratified our descriptive analysis of beliefs by smoking status. Students who had smoked at least once a week in the past 30 days were considered to be regular smokers (11.3%), while the remaining students were classified as “not weekly smokers” (88.7%).

As we hypothesized that the gender context of a country would influence the prevalence of beliefs in each gender, we classified the seven countries according to their gender culture using the Gender Inequality Index. According to the United Nations Development Program, there are three dimensions to the Gender Inequality Index [41]: health (maternal mortality rate and adolescent birth rate), empowerment (female and male population with at least a secondary education, female and male participation in parliament), and labor participation (female and male participation in labor forces). We classified the seven countries according to their scores in the Gender Inequality Index (from the most equal to the least equal): the Netherlands (0.044, 3rd), Finland (0.056, 8th), Germany (0.066, 9th), Belgium (0.073, 12th), Italy (0.085, 16th), Portugal (0.091, 17th), and Ireland (0.127, 26th).

Finally, we controlled our models for other sociodemographic variables, all of which have been found to be related to smoking [42–44]. First, we controlled for age (“How old are you?”, with possible answers between 12 and 18 years of age). There were slight differences between country samples regarding age: the mean age was 15 years in Belgium, Ireland, Finland, Italy, and The Netherlands, but was younger in Germany (14 years old) and older in Portugal (16 years old).

Then, we also controlled for migrant status (“Were you born in this country?” with “yes” or “no” as possible answers). Third, we controlled for the level of education of father and mother: “What was the highest level of schooling your father/mother attended?”, with the possible answers: “completed primary school or less”, “secondary school, not completed” (both grouped into the Low category); “completed secondary school”, “college or university, not completed” (both grouped into the Medium category); “Completed college or university” (High category); and “I don’t know” (Unknown category). Fourth, we controlled for parental smoking: “Does any member of your family smoke cigarettes?”: adolescents with at least one parent (mother or father) currently a smoker received 1 point, other students received 0. Finally, we controlled for socioeconomic status, which was measured using the MacArthur scale [45]: each student was asked to situate his/her own socioeconomic position on a 0–10-point scale, with 0 = the worst socioeconomic situation of an inhabitant of his/her country and 10 = the best socioeconomic situation of an inhabitant of his/her country.

### Statistical analysis

First, we tabulated the description of our sample (Table 2). Then we displayed the frequency of beliefs by gender and by country (Table 3). To do so, we gave the percentage of agreement with each item of the four belief scales and used ANOVA (analysis of variance, F-test and *p*-value) to analyze significant gender differences and country differences. In order to control for multiple testing, we used the Duncan option of the ANOVA procedure (alpha: 0.05). Third, we repeated the same analysis with a stratification by gender and smoking status, in order to identify gender differences in beliefs across smoking statuses (regular smokers versus those who were not regular smokers) (Table 4). Fourth, we ran four multinomial regressions for each individual belief scale and ran a contrast analysis by gender and country, in order to identify whether gender differences in the four belief scales varied significantly between countries (Table 5). Fifth, we repeated this analysis with gender-country interaction and verified whether the interaction coefficient varied when the gender-country Gender Inequality Index was added to the model (results not presented). For all analyses, we used SAS 9.4.

**Table 2:** Socio-demographic description of the sample according to gender. Silne R, 2016, *N* = 12,979

	Gender				
	Girls		Boys		chisq
	% of mean	N	% of mean	N	
Subjective socioeconomic status (mean, /10)	6.7	6293	6.8	6141	39.0**
Age (mean)	14.9	6517	15.08	6452	79.3**
Migration status (%)					0.4
Born in another country	8.4	546	8.7	561	
Born in this country	91.6	5974	91.3	5898	
Country (%)					34.4**
Belgium	14.7	960	15.1	977	
Ireland	16.8	1097	15.8	1018	
Finland	12.9	838	13.9	897	
Germany	11.5	748	11.6	749	
Italy	16.7	1090	13.6	880	
The Netherlands	13.5	877	15.2	984	
Portugal	14	910	14.8	954	
Educational level of mother (%)					40.5**
Low	14.4	940	13.5	871	
Medium	32.4	2112	30.9	1993	
High	39.2	2556	37.9	2449	
Other	0.2	14	0.1	7	
Unknown	12.6	822	15.9	1024	
Missing	1.2	76	1.8	115	
Educational level of father (%)					4.8
Low	17.9	1169	17.4	1123	
Medium	28.3	1847	28.7	1856	
High	35.1	2286	34.4	2223	
Other	0.1	7	0.1	9	
Unknown	17.1	1116	17.5	1128	
Missing	1.5	95	1.9	120	
Parental smoking (%)					11.5**
No parental smoking	84.7	5519	86.7	5602	
At least one parent smoker	15.4	1001	13.3	857	
Weekly smoking of students (%)					0.1
Not weekly smoker	88.6	5778	88.8	5737	
Weekly smoker	11.4	742	11.2	722	

*p < 0.05; ** < 0.001

**Table 3:** Percentage of agreement with each smoking belief, according to gender and country of data collection, analysis of variance (ANOVA, *p* value). Silne R, 2016/17, *N* = 12,979

	BOYS								GIRLS								ALL	ANOVA gender (F)	Chisq for country
	Bel_1_	Ire	Fin	Ger	Ita	NL	Po	ALL	Bel	Ire	Fin	Ger	Ita	NL	Po	ALL			
Positive individual beliefs																			
Feeling relaxed	49	49	28	37	47	50	43	44	49	44	25	36	56	50	39	43	44	0.1	60.6*
Losing weight	22	30	10	15	13	12	22	18	20	33	10	22	16	15	27	21	20	10.7*	67.6*
Positive social beliefs																			
Looking cool	13	18	7	7	19	14	13	13	11	15	7	8	18	14	10	12	13	1.3	31.1*
Looking grown-up	10	33	11	18	20	19	12	18	11	31	9	19	25	18	11	18	18	0.5	89.7*
Looking masculine	8	17	8	12	13	14	9	12	6	15	6	10	7	9	5	8	10	33.5*	25.5*
Looking feminine	2	7	5	4	6	4	3	5	5	3	2	3	8	3	3	4	4	2.9	9.8*
Getting a boy/girlfriend	4	10	6	4	11	6	5	7	3	6	4	4	5	5	4	4	6	29.8*	10.2*
Becoming popular	7	21	11	7	12	15	13	12	9	13	11	10	12	13	14	12	12	1.4	17.4*
Appearing sexy	4	9	3	3	13	5	7	7	4	10	4	3	14	7	6	7	7	2.3	39.7*
Negative individual beliefs																			
Getting facial wrinkles	47	84	82	79	63	70	66	70	56	92	90	81	71	77	77	78	74	93.1*	165.5*
Having less ability to exercise	90	90	88	87	74	82	85	85	90	93	94	83	76	81	86	86	86	1.7	53.7*
Getting lung cancer	94	97	95	94	92	92	95	94	95	98	98	96	93	93	97	96	95	18.3*	14.8*
Having trouble breathing	92	93	91	95	88	91	91	91	93	97	96	95	92	92	95	94	93	37.8*	7.7*
Negative social beliefs																			
Getting into trouble	69	85	89	90	75	66	62	76	69	91	93	89	79	71	68	80	78	27.8*	136.9*
Most people reluctant to date	49	65	75	64	49	67	31	57	52	66	77	61	41	64	28	55	56	4.6*	191.7*
Parents’ disapproval	95	98	98	82	95	96	95	95	96	99	98	81	95	97	98	95	95	4.6*	116.6*
Most people would not hire a smoker to babysit	71	82	88	87	78	81	64	79	72	87	88	88	73	82	63	78	78	0	91.4*
Best friends’ disapproval	57	63	50	52	37	46	59	53	69	79	67	58	49	68	73	67	60	265.1*	64.6*
Teachers’ disapproval	38	90	86	68	62	56	60	66	41	96	76	63	61	61	64	67	66	0.8	288.1*

We controlled for multiple testing using the DUNCAN (alpha = 0.05) option of the ANOVA procedure

_*1*_*Bel* Belgium, *Ire* Ireland, *Fin* Finland, *Ger* Germany, *Ita* Italy, *NL* The Netherlands, *PO* Portugal, *All* All countries

* = *p* < 0.05

**Table 4:** Percentage of agreement with each smoking belief, according to gender and country of data collection, analysis of variance (ANOVA, p value). Silne R, 2016, *N* = 12,979

	BOYS			GIRLS			All			Group comparisons		
	Weekly smokers	Not weekly smokers	all	Weekly smokers	Not weekly smokers	all	Weekly smokers	Not weekly smokers	all	ANOVA gender in weekly smokers (F)	ANOVA gender in not weekly smokers (F)	ANOVA smoking status (all, F)
Positive individual beliefs												
Feeling relaxed	86	38	44	88	38	43	87	38	44	2	1	1364.0*
Losing weight	27	17	18	26	20	21	26	19	20	1	14.3*	47.9*
Positive social beliefs												
Looking cool	17	13	13	14	12	12	16	12	13	2	0	11.9*
Looking grown-up	16	18	18	13	19	18	15	18	18	3	2	12.5*
Looking masculine	15	11	12	5	9	8	10	10	10	48.1*	13.8*	0
Looking feminine	5	4	5	7	4	4	6	4	4	2	6.3*	15.3*
Getting a boy/girlfriend	9	6	7	6	4	4	7	5	6	6.0*	24.1*	10.6*
Becoming popular	9	13	12	8	12	12	9	13	12	2	1	19.8*
Appearing sexy	14	6	7	12	7	7	13	6	7	1	4.9*	103.2*
Negative individual beliefs												
Getting facial wrinkles	48	73	70	59	80	78	54	76	74	15.2*	81.8*	346.8*
Having less ability to exercise	68	87	85	72	88	86	70	88	86	3	1	334.4*
Getting lung cancer	87	95	94	89	97	96	88	96	95	2	17.8*	149.1*
Having trouble breathing	80	93	91	85	96	94	83	94	93	5.6*	41.4*	263.2*
Negative social beliefs												
Getting into trouble	47	80	76	53	83	80	50	81	78	5.4*	24.8*	764.9*
Most people reluctant to date	42	59	57	44	57	55	43	58	56	1	6.8*	115.7*
Parents’ disapproval	85	96	95	88	96	95	86	96	95	3	2	266.4*
Most people would not hire a smoker to babysit	64	80	79	63	81	78	64	80	78	1	0	218.8*
Best friends’ disapproval	20	57	53	25	72	67	22	64	60	6.1*	288.6*	1004.9*
Teachers’ disapproval	46	68	66	52	69	67	49	69	66	4.0*	0	219.1*

Note: we controlled for multiple testing using the DUNCAN test option of the ANOVA procedure

*p < 0,05

**Table 5:** Coefficients of country gender differences for each smoking beliefs scale. Contrast coefficients, 95% IC, country classified according to Gender Index of Inequality from the most equal (The Netherlands) to the least (Ireland), Silne R, 2016, *N* = 12,979

	Gender effect in smoking beliefs (female versus male)												
	Positive individual			Negative individual			Positive social			Negative social			Gini INDEX
	Beta	95% IC		Beta	95% IC		Beta	95% IC		Beta	95% IC		
All countries	0.03	0.00	0.05	0.04**	0.03	0.05	−0.11**	−0.15	−0.07	0.04**	0.03	0.05	
The Netherlands (low Gender Index of Inequality)	0.04	− 0.01	0.10	0.02	−0.01	0.05	− 0.08	− 0.18	0.01	0.07**	0.03	0.10	0.044
Finland	−0.08	− 0.15	− 0.01	0.06**	0.03	0.10	− 0.20**	− 0.32	− 0.08	0.03	− 0.01	0.06	0.056
Germany	0.10	0.03	0.16	0.00	−0.03	0.04	0.05	−0.07	0.17	0.00	−0.04	0.04	0.066
Belgium	−0.03	−0.08	0.02	0.03	0.00	0.07	−0.02	−0.13	0.09	0.06	0.02	0.09	0.073
Italy	0.19**	0.14	0.24	0.05	0.02	0.08	−0.08	−0.17	0.00	0.01	−0.03	0.04	0.085
Portugal	0.00	−0.05	0.05	0.05**	0.02	0.08	−0.20**	−0.31	− 0.10	0.06	0.02	0.09	0.091
Ireland (high Gini)	−0.04	−0.08	0.00	0.04	0.02	0.07	−0.22**	−0.29	− 0.15	0.07**	0.04	0.10	0.127
Model -2LogL	−39,516			−30,042			−21,534			−27,175			

Note: the coefficients are contrast estimates from one model for each belief scale

* = *p* < 0.05; ** = *p* < 0.001

## Results

### Sample description

Of the 12,979 students interviewed in 2016, 50.2% were girls and 8.5% had a migration background (Table 2). The boys were slightly older and of higher socioeconomic status than the girls (mean age = 14.9 for girls and 15.8 for boys; chisq = 79.3; *p* < 0.001; mean SES = 6.7 for girls, 6.8 for boys; chisq = 39.0; *p* < 0.001). About 1 out of 7 students had a least one parent who was a smoker and 11.3% of students were weekly smokers (at least one cigarette per week).

### Gender differences in smoking beliefs

Table 3 shows the frequency of agreement with each smoking belief, by gender and by country. There were some gender differences: for 10 out of 19 smoking beliefs, gender differences were statistically significant, though the magnitude of the difference was mostly about 3–4%, with two exceptions: “smoking is associated with having facial wrinkles” (78% of girls; 70% of boys; *F* = 93; *p* < 0.05) and “my friends would disapprove if I smoked” (67% of girls; 53% of boys; *F* = 265; *p* < 0.05).

Out of the positive individual beliefs, girls were more likely to believe that “smoking helps you to lose weight”. As far as negative individual beliefs were concerned, girls agreed particularly frequently with: “smoking is associated with having facial wrinkles”, “smoking leads to lung cancer”, and “smoking gives you trouble breathing”. Moreover, girls more frequently held negative social beliefs: “my friends would disapprove if I smoked”, “smoking gets you into trouble”, and “my parents would disapprove if I smoked”.

On the other hand, boys were significantly more likely to believe in three social beliefs: that smoking helps you to “look masculine” (12% of boys, 8% of girls; *F* = 33; *p* < 0.05) and “get a boy/girlfriend” (7% of boys; 4% of girls; *F* = 30; *p* < 0.05), both positive beliefs, and that “most people would be reluctant to date a smoker” (57% of boys; 55% pf girls; *F* = 5; *p* < 0.05), a negative belief.

### Country differences in smoking beliefs

Regarding the differences in smoking beliefs between countries, we first acknowledge that all beliefs differed significantly between countries and negative social beliefs displayed the largest F-test coefficients. Teachers’ disapproval was the belief with the most pronounced differences between countries (*F* = 288, *p* < 0.05). The belief that “most people would be reluctant to date a smoker” had the second largest chi-square value (*F* = 1092, *p* < 0.05). The beliefs that smoking “gets you into trouble” and elicits “parents’ disapproval” and that “most people would not hire a smoker to babysit their children” had, respectively, the fourth-, fifth-, and sixth-highest chi-square test values. More precisely, for both genders, “parents’ disapproval of smoking” was low in Germany, the belief that “most people would not hire a smoker to babysit their children” was low in Belgium and Portugal, the “best friend’s disapproval” was low among Italians, and the belief that smoking “gets you into trouble” was low in Portugal.

Aside from these variations in negative social beliefs, one negative individual belief also displayed a large F-test value for differences between countries: “getting facial wrinkles” (*F* = 165, *p* < 0.05). On the opposite end of the scale, positive social beliefs, though they differed significantly between countries, displayed the smallest chi-square test values, particularly for the belief that smoking helps you to “get a boy/girlfriend” (*F* = 10, *p* < 0.05) and the belief that smoking helps you to “look feminine” (*F* = 10, *p* < 0.05).

### Smoking status differences in smoking beliefs across genders

Almost all beliefs (18 out of 19) differed significantly between smoking statuses, and some beliefs were particularly embedded in weekly smokers’ minds: “Do you think that smoking increases your chances of feeling relaxed?”, “my best friend (would) disapprove if I smoke”, and “smoking will get me into trouble”. F-test values were particularly high for the negative social beliefs items. Looking at gender differences within each smoking status, however, we found results similar to those in the total sample (both smoking statuses together).

### Gender differences in smoking beliefs: country contrasts

In order to answer our second hypothesis, we focused on the gender context of these countries, to see whether it was related to smoking beliefs. First and foremost, Germany and Belgium were the only countries with no gender differences in the four belief scales.

In Italy, girls scored significantly higher than boys on the positive individual belief scale (b = 0.19, 95% IC: 0.14–0.24). Moreover, regarding the negative individual belief scale, we found significant gender coefficients in Finland (b = 0.06; 95% IC: 0.03–0.10) and Portugal (b = 0.05; 95% IC: 0.02–0.08). Furthermore, more boys had positive social beliefs in Finland, Portugal, and Ireland, while more girls had negative social beliefs in the Netherlands and Ireland (b = 0.07; 95% IC: 0.03–0.10 for the Netherlands; b = 0.07; 95% IC: 0.04–0.10 for Ireland).

In a supplementary model, not presented here, we looked at the interaction between country and gender in a first step and added, in a second step, the interaction between gender and Gender Inequality Index. These did not significantly improve the model fit, nor did they change country coefficients: this shows that a country’s score in the Gender Inequality Index is not significantly associated with any gender differences in the smoking belief scales.

## Discussion

### Main findings and interpretation

Our study aimed to test whether smoking beliefs among adolescents differ according to gender. We expected girls to score higher on positive individual, negative individual, and negative social beliefs, but postulated that there were no gender differences in positive social beliefs. We found that one out of two smoking beliefs differed significantly between genders, although the magnitude of differences was small, except for two beliefs. More precisely, negative social beliefs were more common in girls, while beliefs about the dating-related aspects of smoking, mostly positive social beliefs, were more common in boys.

In this paper, we have shown that negative social beliefs in particular, such as “getting into trouble” or “friends’ and parents’ disapproval”, were more often held by girls than by boys. This is consistent with Curry’s finding [9] that girls are more influenced by extrinsic motivators, such as friends’ opinions. Similar findings were presented in Grogan’s study [14], which concluded that the most important predictor of a girl’s smoking status at 13 and 15 years of age was her friends’ and family’s negative opinions of smoking 2 years earlier. This also corroborates Cremers and colleagues, who found that intention to smoke among girls was related to low rates of negative beliefs [13]. In contradiction of our hypothesis, however, boys were more likely to hold positive social beliefs, though Cremers and colleagues also pointed out that boys had a greater intention to smoke when they had high levels of positive beliefs [13]. However, as far as positive individual beliefs were concerned, we found that girls were not more likely to believe in the relaxing effects of smoking. They agreed more with statements about the effects of smoking on body weight, however, which suggests a more ambiguous association between girls and the positive aspects of smoking than expected.

Secondly, we hypothesized that countries with a more gender-equal culture would have fewer gender differences in beliefs, particularly in social (positive and negative) smoking beliefs. With regard to this second hypothesis, we found no relation between a country’s score in the Gender Inequality Index and gender differences in smoking belief scales, but we did find some variations between countries. In Belgium and Germany, there were no gender differences in smoking belief scales. Italian girls were significantly more likely to agree with individual positive beliefs than Italian boys. In other countries (the Netherlands, Finland, Portugal, and Ireland), though they had very different scores on the Gender Inequality Index, we found national gender contrasts, with girls scoring more highly for positive individual, negative social, and negative individual beliefs and with boys scoring more highly for positive social beliefs. This contradicts Piko [46], who found that girls were more influenced by the social effects of smoking than boys in countries with a more traditional gender culture, compared to the US [46]. There are two ways in which we might explain the absence of a relation between the Gender Inequality Index and smoking beliefs. Firstly, we might suggest that the Gender Inequality Index score concerns countries as a whole, whereas our data only concerns one city in each country. Secondly, we might consider the components of the Gender Inequality Index and acknowledge how much it focuses on aspects of adult life, not adolescent life. To get closer to the gender context of adolescent life, therefore, it may be necessary to broaden the sample to the whole country and/or look at the gender context within schools, which has been proven to influence gendered substance use in adolescents [47].

### Policy implications

Our results underlined the important potential of negative social beliefs: they displayed the largest gender and country variations and also displayed the largest differences between regular smokers and those who were not regular smokers. Negative aspects of smoking have been core elements of prevention strategies over the last few decades. The health effects of smoking have been broadcast widely but, as argued by Ruiz-Moral [48], the fact that negative outcomes of smoking are mostly long-term risks makes them difficult to use in prevention for adolescents. Our study, however, underlined that negative outcomes can also be short-term, such as “getting into trouble” or friends’ disapproval. These negative social beliefs could thus be key elements of smoking prevention programs in the future, particularly in the Netherlands and Ireland. In these national contexts, therefore, four main negative social beliefs, related to short-term disadvantages of smoking, should be emphasized in girl-specific prevention programs. First, prevention programs could address the fact that some parents would not let a smoker babysit their children, a common form of income for adolescents. Second, knowledge of smoking bans at school and similar sanctions should be reinforced, in order to highlight how smoking at school might get students into trouble. Third, parents should be involved in both preventing and sanctioning smoking and should clearly state their disapproval of smoking. Last but not least, the disapproval of smokers’ friends is also a short-term consequence of smoking and, because friends are particularly important in an adolescent’s life, smokers’ friends could be a factor in smoking prevention. Thus, peer-led interventions to prevent smoking could be particularly effective and further studies should pay attention to gender patterns in these interventions [49].

Nonetheless, boys might also benefit from gender-specific programs. We found that they were more likely to believe in the dating consequences of smoking: they indeed believed that smoking helps you to look masculine and get a girl/boyfriend and were less likely to believe that most people would not date a smoker. Prevention programs should, therefore, address these beliefs in order to reduce the social attractiveness of smoking, particularly in Ireland, Finland, and Portugal, which displayed the largest gender contrasts on this belief scale, with boys scoring more highly. This belief scale displayed, however, lower F-test values between smoking statuses, compared to those of the negative social beliefs scale. The impact of such prevention programs on reducing regular smoking could thus be limited, although further studies need to test for this hypothesis with a longitudinal sample.

Two opposing strategies can be derived from our results. On the one hand, policy-makers might capitalize on these gender differences and reinforce prevention strategies that address negative social beliefs for girls and dating aspects of smoking for boys. There is a risk, however, that this might increase gender differences in beliefs. On the other hand, policy-makers might choose to do the opposite by, for example, reinforcing negative social beliefs in boys. The risk then is that this might not work, if boys are not sensitive to these arguments. Moreover, as we underlined in the introduction, boys’ gender socialization encourages them to break the rules and engage in more deviant behavior. Thus, emphasizing how smoking might get them into trouble or that their parents would disapprove of their smoking might produce the opposite effect and increase their willingness to smoke.

### Limitations

First, this study is cross-sectional, which prevents us from establishing a causal relationship between smoking beliefs and smoking intention. Nonetheless, many studies using the theory of planned behavior [50, 51] have found a direct causal relation between smoking beliefs and later smoking status. Further studies are needed, however, to link our results regarding gender and beliefs to the actual uptake of the behavior. Second, we did not interview students throughout each country, but rather chose a single city in each, which corresponded to the mean socioeconomic situation of the country [52]. Moreover, our study would have benefited from more in-depth analysis of contextual influences on smoking beliefs: more local analyses are needed to understand why particular smoking beliefs are more prevalent in some contexts.

## Conclusions

In this paper, we developed a typology of beliefs that allowed us to make two assumptions. Our hypothesis that smoking beliefs would differ across genders was true for one out of two beliefs. More precisely, negative social beliefs were more frequently held by girls, while beliefs about the dating-related aspects of smoking (mostly positive social beliefs) were more frequently held by boys. To develop gender-specific interventions to target these kinds of beliefs, two opposing strategies might be used, depending on whether such programs are aimed at boys or girls; these might result in counterproductive effects. To reach a conclusion on the influence of negative social beliefs on later gender smoking status, further longitudinal studies are needed. Furthermore, we could not conclude that the gender equality context of a country was associated with these beliefs. Further studies could investigate the influence of more local gender contexts on smoking beliefs, within schools, for example.

## Data Availability

At the time of submission, data were not yet published on a repository. This information would be given once a deposit has been made. Information about dataset and its accessibility can be addressed to the corresponding author.

## Abbreviations

ANOVA: Analysis of variance (statistical procedure)
SILNE R: Smoking Inequalities Learning from Natural Experiments – Renew
